# Single-cell network biology enabling cell-type-resolved disease genetics

**DOI:** 10.1186/s44342-025-00042-7

**Published:** 2025-03-27

**Authors:** Junha Cha, Insuk Lee

**Affiliations:** Department of Biotechnology, College of Life Science and Biotechnology, Yonsei University, Seoul, 03722 Republic of Korea

**Keywords:** Single-cell network biology, Cell-type-specific networks, Cell-type-resolved genetics

## Abstract

Gene network models provide a foundation for graph theory approaches, aiding in the novel discovery of drug targets, disease genes, and genetic mechanisms for various biological functions. Disease genetics must be interpreted within the cellular context of disease-associated cell types, which cannot be achieved with datasets consisting solely of organism-level samples. Single-cell RNA sequencing (scRNA-seq) technology allows computational distinction of cell states which provides a unique opportunity to understand cellular biology that drives disease processes. Importantly, the abundance of cell samples with their transcriptome-wide profile allows the modeling of systemic cell-type-specific gene networks (CGNs), offering insights into gene-cell-disease relationships. In this review, we present reference-based and de novo inference of gene functional interaction networks that we have recently developed using scRNA-seq datasets. We also introduce a compendium of CGNs as a useful resource for cell-type-resolved disease genetics. By leveraging these advances, we envision single-cell network biology as the key approach for mapping the gene-cell-disease axis.

## Introduction

Networks serve as a powerful biological representation of gene properties, as functions are often defined by the relationship between a set of genes such as pathways and gene clusters. Moreover, in addition to understanding each gene’s individual function, mapping the genetic network provides a system-wide perspective on the intricacies of genetic functions and offers insights into each gene’s overall contribution to the system [1]. Gene networks have been successfully applied to infer the functional priority of genes in various diseases [2], and network properties have been found to implicate genetic changes through mutations or epigenetic modifications [3]. However, cellular contexts, including specific cell types involved in disease processes, are essential for accurate network interpretations.

Recent advances in next-generation sequencing (NGS) technology have allowed high throughput transcriptome profiling at low cost, and collective uploading of generated NGS data to curated public repositories [4] has made them easily accessible. With single-cell RNA sequencing (scRNA-seq), where individual cells are measured for their transcriptional activities, gene co-expression within computationally selected cells can serve as a surrogate for inferring genetic associations within specific cellular contexts. The large number of samples provided by scRNA-seq enables the use of linear association metrics such as Pearson correlation coefficients (PCC), or non-linear association metrics such as mutual information [5]. However, spurious and spontaneous expressions in scRNA-seq profiles make data interpretation particularly challenging. Furthermore, each cell typically expresses only a limited subset of genes, and this sparsity is compounded by the imperfect transcript-capturing efficiency of various single-cell platforms [6]. As a result, single-cell datasets are inherently sparse, which creates significant challenges in identifying reliable genetic associations. Consequently, most association metrics have shown limited accuracy across a variety of cellular contexts [7].

One might presume that capturing co-expression using a more dense and “reliable” dataset such as bulk RNA sequencing, would be the better choice. However, inferring gene associations from scRNA-seq data offers a unique opportunity to address cellular context-specific interactions, which is a critical factor for their biological interpretation [8]. It is increasingly clear that the dynamics of genetic relationships are fluid and dependent on a variety of contexts (e.g., cell type). For example, in the context of oropharyngeal cancer, evidence shows that the *KLRB1* gene (encoding CD161) enhances the anti-tumor effect when expressed in CD4^+^ follicular helper cells [9], but functions as an immune suppressor when expressed in CD8^+^ T cells [10, 11]. Furthermore, genetic mutations occur at the cell type-specific level, and compelling evidence suggests that the state of the cell in which these mutations arise may determine the induction of various neurological disorders [12, 13]. This indicates that gene networks must be interpreted in a context-dependent manner and must be modeled in such a way that best represents specific contexts. In this sense, scRNA-seq data provides an excellent opportunity to computationally identify cells of specific states and model their interactions that were previously confounded by compositional differences within tissue-level samples [14].

Networks focusing on gene regulations are well-established, and various methods exist to infer models that explain the regulatory activities of transcription factors and their regulons using scRNA-seq [15]. Functional associations provide a useful generalization of gene interactions that extend beyond transcriptional regulation and allow hypotheses that explain the relationship between genes and their expressions such as epigenetic regulation, expression of quantitative trait loci (eQTLs), and other biological phenomena. Here, we focus on methodologies we have developed mapping the cell-type-specific genetic associations leveraging scRNA-seq datasets and their applications to disease genetics. A reference-based “top-down” approach assumes that interactomes modeled using multiple evidence [16] represent a collection of all possible interactions across a variety of contexts. Essentially, CGNs modeled with this approach are a subset of the interactome representing user-defined cell types. On the other hand, a reference-free approach infers gene associations “bottom-up” and captures associations with the input data only. While the top-down approach infers highly confident associations, it is not suitable for novel discoveries that could be found in a purely data-driven fashion. The bottom-up approach allows de novo interaction detection but must address the abundance of false positive detections. In this review, we focus on specific computational algorithms that we have developed to make both types of inference feasible and explore their applications in cell-type-resolved disease genetics research.

## Reference-based inference of CGNs

**Fig. 1:**
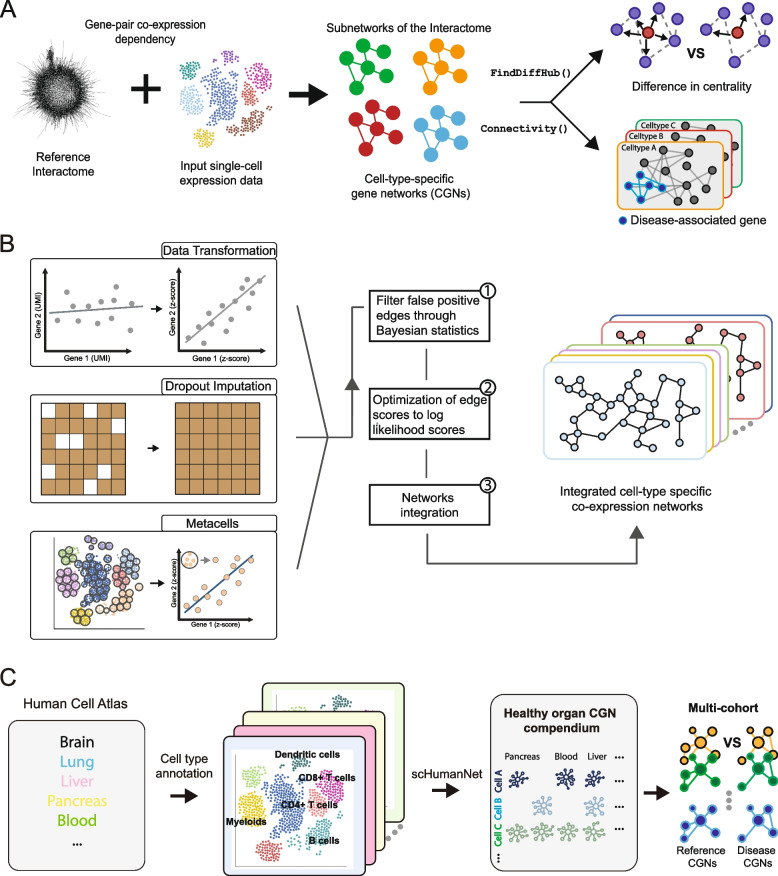
Inference of cell-type-specific gene networks from single-cell transcriptome data and their applications in dissecting disease genetics. **A** scHumanNet framework that infers cell-type-specific networks (CGNs) through filtering reference interactome, HumanNet, and analytical modules for deriving differential hub genes and deconvoluting gene sets based on network connectivity are described. **B** De novo inference of CGNs through various approaches of preprocessing single-cell transcriptome data. The three methods with independent Pearson correlation coefficients (PCC) scores as gene–gene edge weights were converted to log-likelihood score (LLS). High LLS score edges are kept and integrated using the identical approach modeling HumanNet, integrating edges from various sources of evidence. **C** A compendium of reference CGNs inferred from human cell atlas data, demonstrating their utility in identifying disease-associated genes by comparing them with CGNs inferred from disease samples of the same organ

Recently, we have developed scHumanNet (Fig. 1A), a reference-based approach to construct and analyze CGNs [17]. The construction of CGNs depends on the SCINET algorithm [18] with HumanNet interactome [16] selected as the reference scaffold. From scRNA-seq expression data, the SCINET algorithm calculates a gene activity score and converts the skewed distribution of gene expression to a standard normally distributed subspace. For each gene pair existing in HumanNet, the minimum activity score of the two genes within the pair is statistically assessed for its likelihood against the null normal distribution. This is tested for each subsampling of the designated cell types, and each *P*-value from the subsampling is aggregated to a meta-*P*-value using Fisher’s combination method to determine if the interaction exists within the defined cell type. This scheme balances the total number of cells across multiple cell states. When the gene pair is assessed to likely exist within the given cell type, that gene pair becomes part of the CGN with the original weight score of HumanNet.

The validity of the modeled CGNs and their ability to reflect key functional genes are essential for the further application of graph theories and their interpretations in disease contexts. This is not trivial, as there currently exists no consensus on cell-type-specific functional gene sets for model assessment that is regarded as the gold standard. Here we assumed that cell type-associated genes collected from public resources would be distinguished in the modeled CGN topologies, highlighting the networks’ cell-type specificity. We found that when ordering genes (nodes) by their degree centrality values (sum of edge weights), the scHumanNet CGNs significantly better reflected their cell-type specificity compared to other existing methods, for both reference-free and reference-based but with another interactome [19]. This indicates that the reference used as a scaffold for CGN construction greatly affects the performance of the CGNs models and they are generally better at prioritizing prior knowledge-based genes known to have specific expression in cell types. Moreover, this finding additionally supports that the centrality of gene networks is highly associated with central, context-specific biological functions.

Accurate network models also enable various downstream analyses at the cell type-specific level and provide insights into the cellular genetic mechanism of disease. In scHumanNet, we provide a statistical framework that retrieves significant hub genes by comparing multiple CGN models (one versus others). This is achieved by constructing a null model of centrality values through random edge reshuffling. The framework has also been extended to extract differential hub genes within the same cell type between states (e.g., healthy vs. disease). We have found that this method can identify disease genes that exhibit minimal expression changes in the disease state. Moreover, this network-based approach can complement the conventional expression-based approach to prioritize disease genes through differentially expressed genes (DEGs) analysis. Many expression signatures of diseases have been previously proposed based on large-scale transcriptome profiling at the patient level [20]. While these gene sets provide valuable insights for predicting or explaining various clinical outcomes in terms of functional genomics, they are often confounded by various cellular factors existing within the analyzed samples [14]. We found that network structures can be leveraged to deconvolute the cellular context of these programs and help identify cell-type specific mechanisms associated with immunotherapy responses in breast cancer. The scHumanNet framework has also been applied to autism spectrum disorder (ASD) to find the cell type that is most associated with the disease and its respective genes (excitatory and inhibitory neurons), which could not be found through an expression-based approach [21]. Notably, while these genes were not differentially expressed, known ASD-associated genes were prominent in inhibitory neurons—specifically when we focused on those that lost their centrality in ASD CGNs compared to healthy control CGNs. This suggests that changes in network centrality could be interpreted to represent alterations of critical biological properties [22] that are necessary for maintaining health.

## De novo inference of CGNs

While using reference interactions as scaffolds shows great promise, this approach limits the ability to capture de novo gene interactions unique to disease-associated cell types. These previously undiscovered interactions address important gaps in existing knowledge and potentially provide novel therapeutic targets. To make this feasible, we must assess gene expression using a “bottom-up” approach and extract associations directly from the scRNA-seq dataset. Previously, we demonstrated that imputation of gene expression is a viable method for capturing linear gene associations when consequently filtered via the Bayesian framework [23]. In this study, we have validated both computationally and experimentally that this data-driven approach not only facilitates the prioritization of genes associated with breast cancer metastasis but also aids in new target discoveries. During network construction following scRNA-seq data preprocessing, the filtering step retaining only the enriched collection of gene pairs relative to a prior ratio (Bayesian statistics approach using true positive to true negative gene pair ratio) of a well-defined functional gene set is critical [24]. This step controls the numerous false positives that can arise from imputation [25] or other forms of preprocessing such as metacell approaches [26] or count matrix transformations [27].

We have found that unique variations of linear gene association can be obtained depending on the chosen preprocessing method and that some selected methods can retrieve highly functionally enriched gene pairs (Fig. 1B). We have extensively benchmarked existing scRNA-seq preprocessing methods and selected the best-performing approaches based on Bayesian inference, and integrated them to construct the final, comprehensive CGNs [28]. In addition, we hypothesized that de novo interactions modeled from diverse cell types and disease contexts could complement existing reference interactomes in terms of disease gene prediction. Indeed, incorporating over 850,000 newly discovered gene associations through co-expression from multiple scRNA-seq datasets significantly enhanced the prediction performance of the interactome. These findings further confirm the cellular specificity of human disease, the effectiveness of data-driven network inference using single-cell expression data, and its substantial potential to advance precision medicine.

## A compendium of reference CGNs to facilitate the study of gene-cell-disease axis

Recently, cell atlas projects have been initiated for various organs, aiming to establish a “periodic table” of cells from a healthy human body [29]. By leveraging these cell atlases as a reference, it becomes possible to identify differentially expressed genes in each cell type from diseased organs, thereby inferring disease-associated cell types. However, comparisons of gene expression between control and disease samples remain challenging due to the frequent confounding effects of batch variability [30]. Cell harmonization across technologies or batches has been widely applied [31], but methods to harmonize gene expressions across batches in scRNA-seq datasets are still largely limited [32]. Moreover, most single-cell expression datasets investigate cellular heterogeneity in specific contexts (e.g., cancer, viral infection) and often lack suitable controls. Therefore, performing expression-based studies with multiple single-cell datasets remains a major challenge in the field. This suggests that a computational framework designed to leverage independent datasets as controls to prioritize various disease genes would be highly valuable in its utility.

By examining genes with changes in centrality rather than focusing solely on their expression within each cell, we may gain novel functional insights into genes within a specific context. Moreover, this approach may be more robust to batch effects compared to expression-based analyses across datasets [33]. To facilitate this, we may construct a compendium of CGNs inferred from human cell atlas data, serving as a valuable reference database for identifying disease-associated genes through centrality changes between CGNs inferred from disease samples and those corresponding to the same organ in a healthy state. To support this multi-data comparison using network-based approaches, we developed HCNetlas [34], a database of reference CGNs comprising 198 CGNs spanning 61 cell types across 25 human organs (Fig. 1C).

Indeed, we have observed in multiple case studies including systemic lupus erythematosus (SLE), lung cancer, and Alzheimer’s disease that this approach effectively prioritizes known genes associated with the disease while also enabling new discoveries. Importantly, we compared differential centralities using the CGNs inferred from in-house healthy control samples of the SLE public dataset [35] and observed highly similar results when using the reference CGNs from the compendium. This indicated that in the context of network centrality comparisons, control cells from independent datasets (of the same cell type) can effectively be leveraged for downstream analyses to understand gene-cell-disease relationships.

## Integration and future outlook

In this review, we introduced several computational frameworks and resources we recently developed in the field of single-cell network biology. With only scRNA-seq data as input, we outline valid approaches to construct cell-type-specific gene association networks, both reference-based and reference-free. We also present a resource of databases and analysis methods designed to leverage networks for gene prioritizations within any specific context of single-cell gene expression. In multiple case studies, we have demonstrated that network topology analysis can reveal new information that could not be retrieved via conventional DEG analysis. Moreover, we show that network topology at the cell type-specific level can elucidate cellular mechanisms underlying various disease signatures previously identified through patient-level studies. Lastly, we report that network-based approaches may serve as a potential framework for addressing technical variations in gene studies conducted with multiple scRNA-seq datasets. Our research provides valuable insights and represents key potential advances in the field of network medicine.

Most algorithmic developments for biological networks have previously focused on achieving high accuracy in modeling cellular networks. With scRNA-seq datasets in particular, emphasis has been placed on various ways to infer gene interactions and construct gold standards to make informed assessments of the network, as this remains a non-trivial task [36, 37]. We note that as there is a lack of consensus in assessing the accuracy of cell type-specific networks in various contexts, hypotheses driven by network structures must be cautiously performed with networks of sufficient accuracy. Assuming a well-modeled CGN in a specific context creates new possibilities specifically in disease genetics. For example, such networks may be utilized as foundational structures to train a graph neural network for tasks suited to understanding cellular mechanisms of disease [38]. Additionally, various graph theories such as percolation theory or attack/failure analysis to evaluate how CGNs behave under node or edge addition/removal should be explored for use as network-based in silico knock-in and knockout experiments. Ultimately, well-modeled CGNs and their analysis will allow cell-type-resolved disease gene identification, a primary endpoint in disease genetics.

We have found potential evidence suggesting that network structures may be more robust to scRNA-seq batch effects, an intriguing observation that could be developed further. Rigorous testing across various datasets with a wide range of batch effects should be conducted to assess whether network structures maintain structural similarity across batches of similar contexts. If so, differential network analysis may emerge as a valuable tool for performing gene-level meta-analysis across multiple scRNA seq datasets, a task that is currently challenging due to computational limits. Additionally, genetic properties that are associated with networks must be further investigated. For example, it has been recognized that gene isolation has a significant effect on ASD phenotype without any corresponding expressional changes. Genes with changes in network centrality but not in expression level in matched disease samples could potentially reflect pathogenesis. This hypothesis could be further explored in the future through the integration of multi-omics datasets, such as examining whether expression variances and network topological changes align with cell-type-specific mutations or chromatin accessibility. Systematic exploration of gene-cell-disease relationships through multi-omic CGNs holds immense potential for advancing precision medicine. Lastly, computationally collecting a variety of “similar” state cells and constructing networks based on their heterogeneity shares conceptual similarities with pseudo-bulk methods. A natural question arises regarding the adequate resolution needed for an interpretable network. This could be evaluated in the future using experimentally validated cellular gold standard gene associations that will necessarily be needed for future CGN constructions with various resolutions.

## Data Availability

No datasets were generated or analysed during the current study.

## Abbreviations

scRNA-seq: Single-cell RNA sequencing
CGNs: Cell-type-specific gene networks
NGS: Next-generation sequencing
PCC: Pearson correlation coefficient
eQTLs: Expression quantitative trait loci
DEGs: Differentially expressed genes
ASD: Autism spectrum disorder
SLE: Systemic lupus erythematosus
